# Anosognosia in hoarding disorder is predicted by alterations in cognitive and inhibitory control

**DOI:** 10.1038/s41598-022-25532-4

**Published:** 2022-12-16

**Authors:** Peter J. van Roessel, Cassandra Marzke, Andrea D. Varias, Pavithra Mukunda, Sepehr Asgari, Catherine Sanchez, Hanyang Shen, Booil Jo, Lisa A. Gunaydin, Leanne M. Williams, Carolyn I. Rodriguez

**Affiliations:** 1 Department of Psychiatry and Behavioral Sciences, Stanford University School of Medicine, 401 Quarry Road, Stanford, CA 94305 USA; 2 Sierra Pacific Mental Illness Research, Education, and Clinical Center (MIRECC), Veterans Affairs Palo Alto Health Care System, Palo Alto, CA 94304 USA; 3 Department of Psychiatry and Behavioral Sciences, University of California San Francisco, San Francisco, CA 94143 USA; 4 Touro University College of Osteopathic Medicine, Vallejo, CA 94592 USA; 5 Department of Epidemiology and Population Health, Stanford University School of Medicine, Stanford, CA 94305 USA; 6 Kavli Institute for Fundamental Neuroscience, University of California San Francisco, San Francisco, CA 94143 USA; 7 Veterans Affairs Palo Alto Health Care System, Palo Alto, CA 94304 USA

**Keywords:** Disorders of consciousness, Psychiatric disorders

## Abstract

Insight impairment contributes significantly to morbidity in psychiatric disorders. The neurologic concept of anosognosia, reflecting deficits in metacognitive awareness of illness, is increasingly understood as relevant to psychopathology, but has been little explored in psychiatric disorders other than schizophrenia. We explored anosognosia as an aspect of insight impairment in *n* = 71 individuals with DSM-5 hoarding disorder. We used a standardized clutter severity measure to assess whether individuals with hoarding disorder underreport home clutter levels relative to independent examiners. We then explored whether underreporting, as a proxy for anosognosia, is predicted by clinical or neurocognitive behavioral measures. We found that individuals with hoarding disorder underreport their clutter, and that underreporting is predicted by objective severity of clutter. In an *n* = 53 subset of participants, we found that underreporting is predicted by altered performance on tests of cognitive control and inhibition, specifically Go/No-Go and Stroop tests. The relation of underreporting to objective clutter, the cardinal symptom of hoarding disorder, suggests that anosognosia may reflect core pathophysiology of the disorder. The neurocognitive predictors of clutter underreporting suggest that anosognosia in hoarding disorder shares a neural basis with metacognitive awareness deficits in other neuropsychiatric disorders and that executive anosognosia may be a transdiagnostic manifestation of psychopathology.

## Introduction

In the psychiatric literature, ‘insight’ is a complex, multidimensional construct, defined to include awareness of illness, attribution of symptoms to illness, and openness to treatment^1,2^. It is nonetheless of *prima facie* clinical relevance, influencing patients’ engagement with and benefit from care. Across diverse disorders, impaired insight has been associated with increased symptom severity, greater comorbidity, and worse prognosis^3,4^. Insight has been identified as a critical target of psychotherapeutic treatment^5^, yet there is minimal evidence to support pharmacologic or somatic treatments for impaired insight, likely reflecting the dimensional complexity of the construct, the heterogeneity of conditions in which insight may be impaired, and insufficient understanding of the basis of insight in the brain. Pathophysiologic understanding of insight in psychiatry has further emphasized its dynamic psychological nature, including affective and motivational influences on awareness and the deployment of denial as a defense^6^.

In the neurologic literature, impaired awareness of illness—or anosognosia—has been a focus of interest for more than a century and has been described in association with diverse disorders, including most characteristically hemiplegia after stroke, but also cortical blindness, dementias, aphasias, and amnestic disorders^7,8^. In contrast to the dynamic psychiatric model of ‘insight impairment,’ the term ‘anosognosia’ refers specifically to impaired awareness of illness and its symptoms. Pathophysiologic understanding of anosognosia has emphasized its neurobiological basis—the disruption to relevant cortical and subcortical circuits and substrates—particularly implicating right hemispheric areas, including prefrontal, temporoparietal, and insular cortex^9–13^.

The concept of anosognosia has increasingly been applied in the context of psychiatric illness^4^. While in psychiatry the term ‘anosognosia’ has been used principally in studies of schizophrenia^14–16^, research exploring the neurobiological basis of awareness deficits in psychiatric illness has increasingly supported a model in which insight reflects neurobiologically-based neurocognitive capacities. In particular, studies in schizophrenia, substance use disorders, and a limited number of other conditions have suggested that deficits in illness awareness correlate neurocognitively with deficits in aspects of executive function, particularly those related to attentional set shifting and the processing of error^4,17,18^. At a circuit level, awareness or insight deficits have been correlated with dysfunctional activation or connectivity of salience network components, including the anterior cingulate and insula^4,18–20^. In both the psychiatric and neurologic clinical literature, increasing emphasis on anosognosia/insight impairment as reflecting deficits in metacognitive capacity may bridge historical psychologic and neurologic explanatory models^21,22^. A contemporary neurocognitive model for metacognitive awareness, the Cognitive Awareness Model (CAM), similarly emphasizes the role of error-monitoring and other frontal-executive functions in facilitating awareness^23,24^.

In this study, we have sought to explore the neurocognitive basis of anosognosia in psychiatric illness using hoarding disorder (HD) as a highly relevant model disorder. Acquiring and saving behavior leading to excessive and impairing clutter is termed hoarding, and a diagnosis of HD is made when these behaviors are not better explained by a primary neurodegenerative, psychotic, or other condition^25^. Clinical literature has long emphasized manifestations of poor insight in HD^26,27^, and insight impairment is coded as a diagnostic specifier for HD in the Diagnostic and Statistical Manual of the American Psychiatric Association (DSM-5)^25^, yet significant gaps remain in understanding its disorder-specific expression, prevalence, and etiology.

No consensus instrument for assessment of insight has been applied in HD. Scores on the Brown Assessment of Beliefs Scale (BABS)^28^—an insight assessment instrument validated in obsessive–compulsive and related disorders (OCRDs)—correlate poorly with clinician-assessed insight in HD^29^. Attempts to assess the prevalence and degree of insight impairment in HD have yielded inconsistent results, likely reflecting the absence of a standardized assessment, variable samples of the HD patient population, and the inherent limitations of self-report. Studies using clinician interview^30^ or those asking family members^31^ or social service providers^32^ to rate the insight of individuals with hoarding behaviors have suggested rates of poor or absent insight from 55 to 85%. The historical conflation of HD and obsessive–compulsive disorder (OCD) additionally complicates estimates, yet multiple studies have described an association in OCD between hoarding symptoms and insight impairment, whether assessed via the Y-BOCS^33–35^ or BABS^36–38^.

Neurocognitive testing studies in HD have suggested that executive dysfunction, including difficulties with response inhibition and set shifting, is characteristic of the disorder^39–43^. Findings are inconsistent, however: a large study of unmedicated individuals with HD found deficits in sustained attention but no difference from controls in terms of executive function^44^. Neurophysiologic and imaging data is less abundant, yet aberrant activations of anterior cingulate cortex^45^, frontal hypoactivation during inhibitory control tasks^46^, and decreases in an electrophysiologic measure of error processing^47^ have been observed in individuals with HD relative to controls, broadly supporting a model of prefrontal dysfunction in HD. No study has explored the relationship between neurocognitive functioning and insight in HD.

In this exploratory study, we assessed data from individuals screening for participation in a clinical trial of group therapy for HD (NCT02843308) for objective indicators of anosognosia and its correlates. In particular, given that anosognosia, or minimization of clutter, has been considered a hallmark of insight impairment in HD^26,27^, we used a standardized pictographic rating scale of clutter, the Clutter Image Rating (CIR)^48^, to explore individual-level discrepancies between self-ratings of clutter made in clinic and independent examiner (IE) ratings of clutter made during a subsequent home visit. We hypothesized that subjective underreporting of clutter per CIR would be prevalent, and that underreporting would be predicted by impaired performance on neurocognitive tasks associated with attentional control or response inhibition.

## Materials and methods

This study was conducted via an outpatient academic clinic. Approval was obtained for all procedures from the Stanford University Administrative Panel on Human Subjects in Medical Research (authorized to approve research under the US Department of Health and Human Services’ Office for Human Research Protections), and all participants gave informed consent. All experiments were performed in accordance with relevant guidelines and regulations.

### Overview and behavioral assessment of anosognosia

**Figure 1:**
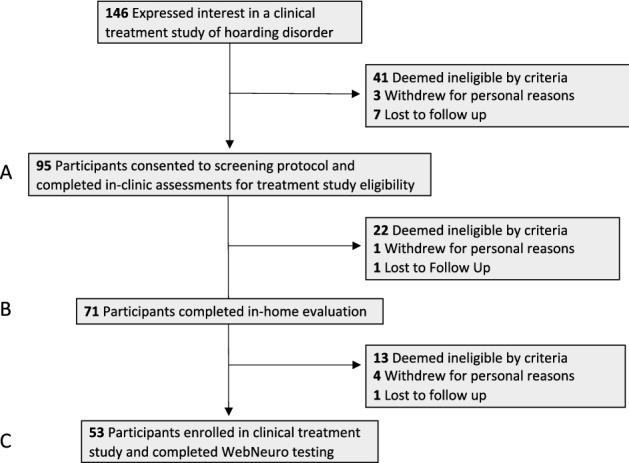
Participant flow diagram. Data were collected from individuals screening for and enrolling in study offering clinical treatment of hoarding disorder. (**A**) Participants underwent clinical assessments and self-rated their home clutter levels using the Clutter Image Rating (CIR). (**B**) 71 participants completed an in-home evaluation during which independent evaluators (IEs) rated home clutter levels using the CIR, thus allowing a measure of anosognosia based upon the relative discrepancy of self- vs IE-rated clutter. (**C**) 53 participants for whom a measure of anosognosia was obtained proceeded to the clinical treatment study and completed computer-based neurocognitive testing (WebNeuro).

Demographic and clinical data were collected from participants screening for participation in a clinical treatment study of HD between October 2016 and April 2019 (Fig. 1A). Participants self-identified as seeking help with clutter and were recruited via targeted online advertisements, radio advertisements, local flyering, and word of mouth. Clinical data included self- and clinician-rated assessments performed either in clinic, via secure teleconference, or via secure web-based submission portals. After initial assessments, a home visit was scheduled during which an IE rated home clutter severity (Fig. 1B). The full *n* = 71 study sample includes all participants for whom both self- and home-visit IE-ratings of clutter were completed during the recruitment period.

A quantitative behavioral measure of anosognosia was generated by evaluating the discrepancy between self-ratings of clutter made using the CIR, a validated pictographic instrument^48^, and objective (IE) ratings of clutter using the same instrument, as a proportion of total objective clutter score (see “Assessments” and “Statistical analysis” below).

### Inclusion/exclusion

To qualify for a home visit (and thus to be included in the *n* = 71 study sample), participants were required to meet the following inclusion criteria: age between 18–75; primary DSM-5 HD diagnosis (assessed via the structured clinical interview (SCID) for DSM-5)^49^; clinically significant symptoms (defined as a Saving Inventory-Revised (SI-R) score ≥ 40)^50^; no or stable psychotropic medication use (defined as medication doses unchanged for > 4 weeks prior to assessment or > 8 weeks if fluoxetine); a safely accessible home in one of two neighboring counties and within 30 miles of Stanford University. Participants were excluded if they had OCD as a primary diagnosis, a current or history of psychotic disorder or bipolar disorder, a current eating disorder, or a current moderate or lifetime severe substance use disorder. Patients were additionally excluded if they had current severe depression (defined as Hamilton Depression Rating Scale (HDRS) score > 30)^51^, had any other medical or neuropsychiatric condition that would increase risk of participation or interfere with engagement in assigned behavioral practice tasks, were currently working with a professional organizer, or were at elevated acute eviction risk. For one participant, a full SCID was completed only after the home visit occurred; however, given the availability of both self- and IE-CIR ratings data, this individual was included in the analysis despite bipolar disorder comorbidity. Similarly, two participants who for scheduling reasons underwent a home visit prior to completing the SI-R, and who were found to have SI-R < 40 (with scores of 14 and 37), were nonetheless included in the analysis given the availability of both self- and IE-CIR ratings and diagnosis of HD per SCID.

### Assessments

The CIR^48^ is a validated pictorial scale of clutter severity consisting of nine photographs each of an increasingly cluttered kitchen, bedroom, or living room. Each room in a home is rated by choosing the image that most closely corresponds to the level of clutter in the room, and a composite home score is generated by averaging room scores. The CIR is designed for use by both patients and clinicians. As validated, the composite CIR score has high test–retest reliability (*r* = 0.85 when repeated by patients with interval of < 2 months) and high inter-observer reliability (*r* = 0.94) when both patient and clinician are in the home being rated; correlation between asynchronous patient ratings made in the clinic and clinician ratings made in the home is less strong (*r* = 0.78)^48^, as similarly observed in an independent study of older adults (*r* = 0.54)^52^. Our data included asynchronous participant CIR ratings (self-CIR) made in clinic and independent examiner CIR ratings (IE-CIR) made during a subsequent home visit. Composite scores for both self-CIR and IE-CIR were calculated by averaging scores from only those rooms rated by both participant and IE.

The SI-R^50^ is a 23-item questionnaire using a 5 point (0 to 4) Likert-type scale that assesses severity of compulsive hoarding. It comprises three subscales defined by exploratory factor analysis, reflecting core domains of hoarding behavior, including Excessive Acquisition (seven items), Difficulty Discarding (seven items), and clutter (nine items). Internal consistency (Cronbach’s alpha) for the full scale in our sample was acceptable, at 0.86. Alpha values for the subscales of the SI-R were 0.7 for Difficulty Discarding, 0.88 for Clutter, and 0.79 for Excessive Acquisition.

The Saving Cognitions Inventory (SCI)^53^ is a 24-item questionnaire using a 7 point (1 to 7) Likert-type scale that assesses the experienced frequency of thoughts and beliefs related to possessions (e.g., “I am responsible for the well-being of this possession”). Although a measure of beliefs, not of hoarding behaviors per se, the SCI correlates strongly with measures of hoarding^54^. Factor analysis of SCI responses has defined four subscales, including emotional attachment (ten items), memory (five items), control (three items), and responsibility (six items)^53^. In our sample, internal consistency of the full scale was high (0.92). Alpha values for the subscales were 0.93 for Emotional Attachment, 0.81 for Memory, 0.68 for Control, and 0.67 for Responsibility.

Additional measures included the 17-item Hamilton Depression Rating Scale (HDRS)^51^, used to allow clinician-assessed rating of depression severity; the North American Adult Reading Test (NAART)^55^, used to estimate verbal intelligence; and the Edinburgh Handedness Inventory^56^ in a revised eight-item format^57^, used to assess handedness.

### Neurocognitive testing

A subset of participants (*n* = 53) met criteria for clinical study enrollment and underwent neurocognitive assessment (Fig. 1C). These criteria included having at least one room IE-CIR rated ≥ 3 on home visit, and having a home deemed safe for ongoing visitation by study staff (i.e., free of mold, vermin, or structural risks).

Neurocognitive assessment of cognitive control was undertaken using a computerized test battery called WebNeuro^58^, which has been validated against gold-standard neuropsychological tests assessing the equivalent constructs^58,59^. The testing battery was completed in a single sitting of approximately 45 min. Participants were offered the opportunity to complete WebNeuro testing either at home or in a private office in the clinic. The neurocognitive domains assessed by this battery and the test used to assess them (and the equivalent test from traditional paper–pencil neuropsychological tests) include:

i. Sensorimotor function; Finger Tapping test (Finger Tapping)
ii. Cognitive control; Go/No-Go test (Go/No-Go)
iii. Maintenance of attention; Continuous Performance test (Continuous Performance)
iv. Processing speed; Switching of Attention test (Trails B)
v. Decision speed; Choice Reaction Time test (Corsi Blocks)
vi. Verbal learning and memory; Memory Recognition test (California Verbal Learning and Memory test)
vii. Working memory; Digit Span test (Digit Span)
viii. Executive function; Maze test to assess planning and response monitoring (Austin Maze)
ix. Inhibition; Verbal Interference task assessing capacity to suppress conflicting information when assessing either written word or ink color (Stroop).

Individual performance on each of these behavioral tests was quantified by accuracy and reaction time relative to matched healthy norms for age, sex, and years of education (normative cohort of *n* = 1317)^60,61^. These norm-reference scores were expressed as standardized scores with a mean of 0 and standard deviation of 1, such that lower scores reflected greater impairment.

### Statistical analysis

Using self- and IE-rated composite CIR scores, we generated an error metric (‘CIR-error’) for each participant that reflected the degree of under- or over-reporting. Taking the IE-CIR as ‘objective,’ we defined CIR-error as self-CIR subtracted from IE-CIR, divided by IE-CIR ((IE-CIR–self-CIR)/IE-CIR), thus capturing discrepancy in self-report in proportion to objective clutter severity. As defined, more positive scores of CIR-error reflect greater underreporting of clutter.

Means, ranges, and standard deviations were assessed for demographic, clinical, and neurocognitive variables. One-sample Student’s t-tests with *µ* = 0 were used to assess differences in neurocognitive performance between our HD participants and the normative population. For those variables for which our participants’ scores were not normally distributed, as per Shapiro-Wilk test, we employed a one-sample Wilcoxon Rank Sum Test. An initial alpha threshold of 0.05 was Bonferroni-adjusted for the number of independent predictors.

We assessed relationships between clinical and neurocognitive variables and CIR-error, as well as self- and IE-CIR scores themselves, using univariate linear regression with CIR-error as the dependent variable. To account for multiple comparisons, the alpha threshold was Bonferroni-adjusted for the number of independent predictors tested. Significant predictors of CIR-error were considered as possible explanatory variables in a multiple linear regression model. Proportions of variance explained are provided as adjusted *R*^2^. Instances of missing data were addressed with listwise deletion; given limited missing observations (< 4 cases per variable), the assumption that data are missing completely at random was not felt to add significant risk of bias. All statistical assessments were performed using R version 4.1.2.

## Results

### Sample characteristics

A demographic and clinical description of our sample population is presented in Table 1. Participants ranged in age from 24 to 75, with an average age of 57.0 (SD 10.4). Fifty-three of 71 participants (75%) in our full sample identified as female and 75% as Caucasian/White. Forty participants (60%) reported use of psychotropic medication, including 36 (54%) using antidepressant medications, and 3 (5%) using antipsychotic medications. The average SI-R score was 59.1 (range 14–86, SD 10.9), well above the thresholds of 39 or 41 suggested in the literature for diagnosis of HD^62,63^. Average HDRS score was 5.8 (range 0–24, SD 4.9), consistent with typically mild severity, if any, of comorbid depression. Estimated verbal intelligence quotient for the full sample was high, at 115.2 (84.1–123.9, SD 7.3), and school grades completed, recorded in the sample completing neurocognitive testing, suggest high levels of education, with 91% reporting at least 4 years of post-secondary education, and 47% reporting more than 6 years of post-secondary education. Medical comorbidity was common, with 65% reporting use of medication for somatic medical conditions.

**Table 1:** Hoarding Disorder Participant Characteristics.

Demographic and clinical variables	Full sample (*n* = 71)	Neurocognitive testing subset (*n* = 53)
Age, *mean (range,* ± *SD)*	57.0 (24–75; ± 10.4)	57.0 (24–75; ± 10.4)
**Gender**^**a**^**, *n* (%)**		
Female	53 (75)	41 (77)
Male	18 (25)	12 (23)
Non-binary/other	0 (0)	0 (0)
**Race**^**a**^**, *n* (%)**		
Caucasian/White	53 (75)	41 (77)
African American/Black	3 (4)	1 (2)
Asian	10 (14)	7 (13)
Native Hawaiian/Pacific Islander	1 (1)	1 (2)
Other/Did not endorse	4 (6)	3 (6)
**Ethnicity**^**a**^**, *n* (%)**		
Hispanic	3 (4)	2 (4)
Non-Hispanic	67 (94)	50 (94)
Did not endorse	1 (1)	1 (2)
**DSM-5 comorbidity (current), *n* (%)**		
Other obsessive-compulsive and related disorder	5 (7.04)	5 (9.43)
Depressive disorder	7 (9.86)	6 (11.32)
Anxiety disorder	6 (8.45)	6 (11.32)
Attention-deficit/hyperactivity disorder	9 (12.68)	9 (17)
Trauma- and stressor-related disorder	4 (5.63)	4 (7.55)
Sleep–wake disorders	9 (12.68)	9 (17)
Other^b^	6 (8.45)	6 (11.32)
**Psychotropic medication use*, n* (%)**		
Any psychotropic medication	40 (60)	28 (53)
Antidepressant	36 (54)	26 (49)
Antipsychotic	3 (5)	3 (6)
**Somatic medical comorbidity**		
Current medical diagnoses^c^, (*mean* (*range;* ± *SD*))	2.62 (0–8; ± 1.90)	2.57 (0–7; ± 1.82)
Any non-psychotropic medication, *n* (%)	42 (65)	31 (65)
**Clinical measures**, **mean (range; ± SD)**		
Saving Inventory-Revised total	59.1 (14–86; ± 10.9)	59.9 (42–86; ± 9.9)
Clutter Image Rating room average, self-rated	3.78 (1.67–6.40; ± 1.14)	3.67 (1.67–6.4; ± 1.12)
Clutter Image Rating room average, independent evaluator-rated	4.22 (1–8.33; ± 1.39)	4.11 (1–6.33; ± 1.17)
Saving Cognitions Inventory total	98.2 (52–150; ± 27.4)	97.5 (52–160; ± 27.2)
Hamilton Depression Rating Scale -17	5.8 (0–24; ± 4.9)	4.8 (0–12; ± 4.0)
Edinburgh Handedness Inventory—laterality quotient	77.9 (-100–100; ± 45.3)	77.3 (-100–100; ± 48.9)
North American Adult Reading Test	115.2 (84.1–123.9; ± 7.3)	116.4 (84.1–123.9; ± 6.1)

^a^Self-asserted. ^b^‘Other’ includes somatic symptom disorder (n = 3), substance use disorder (n = 2), bipolar and related disorders (n = 1). ^c^Self-reported diagnoses include the following categories/conditions (% of full sample): endocrine/metabolic (42%); cardiovascular (29%); musculoskeletal (23%), respiratory/allergic (22%), obstructive sleep apnea (17%); neurologic (16%); remitted cancer (14%); gastrointestinal (14%); urogenital (9%); rheumatologic (4%); dermatologic (3%); hematologic (3%).

### Subjective underreporting of clutter

**Figure 2:**
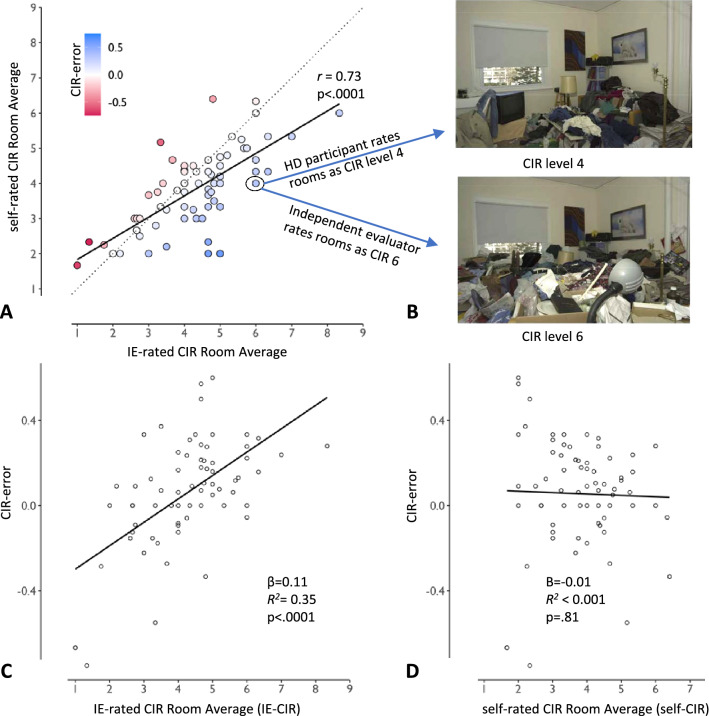
Underreporting of clutter, a proxy for anosognosia, is common in hoarding disorder and increases with severity of clutter. (**A**) Independent evaluator (IE) and self-rated Clutter Image Rating (CIR) scores are correlated (solid black line), but most individuals with hoarding disorder underrate clutter relative to IE ratings (e.g., all points below the x = y dotted line). CIR-error (shading, calculated as (IE-CIR – self-CIR)/IE-CIR) represents the degree of underreporting (blue) or overreporting (red) relative to IE-CIR score. (**B**) Example living room images from the CIR scale (adapted with permission from Steketee and Frost^79^). The circled data point in panel A represents one individual with HD who self-rated their rooms on average as level ‘4’ (upper panel), whereas an IE rated the same rooms on average as level ‘6’ (lower panel). (**C**) Clutter underreporting (CIR-error) increases with objective clutter score (IE-CIR). (**D**) Clutter underreporting is not predicted by self-reported clutter (self-CIR).

Self-assessed composite CIR scores averaged 3.78 (range 1.67–6.4, SD 1.14). IE-CIR scores were obtained during home visit after an average interval of 25 days (range 0–100, SD 21.5). IE-CIR scores averaged 4.22 (range 1–8.33, SD 1.39). Although self-CIR and IE-CIR scores were significantly correlated (*r* = 0.73 (95% CI 0.60–0.83), *p* < .0001), self-CIR scores were significantly lower than IE-CIR scores (difference in means 0.45 (95% CI 0.22–0.67, *t(70)* = 3.94,* p* = .0002), indicating a tendency by participants to underrate clutter during in-clinic assessment (Fig. 2A,B). The mean CIR-error score was 0.06 (range -0.75–0.60, SD 0.26) with 41 of 71 participants (58%) having positive CIR-error values (indicating underreporting of clutter), 21 of 71 participants (30%) having CIR-error score or 0.2 or greater, and three of 71 individuals (4%) scoring 0.5 or greater, representing underreporting of clutter by half.

### Relation of underreporting to clutter severity

Univariate linear regression was used to assess IE-CIR and self-CIR as predictors of CIR-error (Table 2). CIR-error was predicted by severity of IE-assessed clutter (IE-CIR; *β* = 0.11 (95% CI 0.07–0.15), *R*^2^ = 0.35, *p* < 0.0001); the degree of clutter underreporting increased as objective clutter severity increased (Fig. 2C). This was not true for self-reported clutter: self-CIR score did not predict CIR-error (*β* =  − 0.01 (95% CI − 0.06–0.05), *R*^2^ < 0.001, *p* = 0.81; Fig. 2D). The days elapsed between self-rating and in-home IE-rating also had no predictive value (*β* = 0.001 (95% CI − 0.002–0.004), *R*^2^ = 0.012, *p* = 0.37). Using a multivariate linear model, objective severity of clutter (IE-CIR) remained a significant predictor of CIR-error when controlling for age, gender, handedness, estimated IQ, depression, and use of psychotropic medication (*β* = 0.12 (95% CI 0.07–0.16), *p* < 0.0001).

**Table 2:** Demographic and clinical variables as predictors of clutter underreporting (CIR-error).

Demographic and clinical variables (N = 71)	As predictor of CIR-error	
	*β*	*p*^a^
Age	0.004	0.22
Gender	0.000	0.99
Any psychotropic medication use	− 0.098	0.13
Clutter Image Rating room average, self-rated	− 0.007	0.81
Clutter Image Rating room average, independent evaluator-rated	0.110	< 0.0001*
Saving Inventory-Revised (SI-R) total	− 0.003	0.31
SI-R Difficulty Discarding subscale	− 0.018	**0.02**
SI-R Excessive Acquisition subscale	− 0.006	0.36
SI-R Clutter subscale	0.002	0.68
Saving Cognitions Inventory (SCI) total	− 0.001	0.36
SCI Emotional Attachment subscale	0.001	0.80
SCI Responsibility Subscale	0.002	0.68
SCI Memory subscale	0.000	0.99
SCI Control subscale	− 0.009	0.27
Hamilton Depression Rating Scale 17	0.006	0.40
Edinburgh Handedness Inventory—laterality quotient	0.001	0.11
North American Adult Reading Test	0.001	0.78

^a^*p* values < 0.05 are indicated in bold text.

*Meets Bonferroni-corrected alpha threshold of *p* < 0.003.

### Relation of clutter underreporting to clinical assessments

Other demographic and clinical variables were next assessed as predictors of CIR-error (Table 2). For the full sample, CIR-error was not predicted by age, gender, handedness, psychotropic medication use, number of current somatic medical diagnoses, or depression (HDRS). Notably, clinical measures of HD were also not predictive of CIR-error. Only the Difficulty Discarding subscale of the SI-R (SIR-DD) showed a trend toward prediction of CIR-error—however, with a negative coefficient, such that lower scores on SIR-DD predicted greater underreporting of clutter (*β* =  − 0.02 (95% CI − 0.033 to  − 0.003), *R*^2^ = 0.08, *p* = 0.021). The SCI and its component subscales were similarly not predictive of CIR-error.

### Neurocognitive performance

Fifty-three participants completed computer-based neurocognitive testing. Demographic and clinical characteristics of this subset were like those of the full sample (Table 1). Means for normed performance on task measures are represented in Table 3. Our participants with HD showed significant differences from the reference normative population on several measures. On the Go/No-Go test, participants had slower reaction times (*z* =  − 1.08, *t*(51) =  − 14.9, *p* < 0.0001). On the Continuous Performance test, participants showed slower reaction times (*z* =  − 1.01, *t* =  − 6.3, *p* < 0.0001) and a trend toward more false negative errors (*z* =  − 0.38, *V* = 495, *p* = 0.051), suggesting mild deficits in sustained attention. Participants showed stronger performance on a test of immediate recall than the reference population (*z* = 0.49, *V* = 1236, *p* < 0.0001). On the Stroop-like Verbal Interference test, HD participants were marginally faster at identifying the ink color of printed color words (*z* = 0.29, *t* = 2.13, *p* = 0.04), and demonstrated faster normed performance for ink-color identification relative to written color word identification (*z* = 0.24, *t* = 2.32, *p* = 0.02). HD participant performance did not otherwise differ from the reference population for measures of psychomotor speed, reaction time, Switching of Attention, Digit Span, or Maze completion.

**Table 3:** Neurocognitive performance of hoarding disorder participants relative to normative population.

Neurocognitive variables (N = 53)	Mean Z-score (range, ± SEM)	One-sample test statistic (*µ* = 0)	*p* value^a^
Finger Tapping speed	− 0.073 (− 4.33 to 2.70, ± 0.179)	t(49) = − 0.40863	0.685
Go/No-Go reaction time	− 1.076 (− 2.43 to 0.19, ± 0.072)	t(51) = − 14.858	< 0.0001*
Go/No-Go false alarm (no-go) errors	0.200 (− 1.63 to 2.33, ± 0.129)	V = 791	0.3553
Go/No-Go false miss (go) errors	− 0.187 (− 3.28 to 0.95, ± 0.164)	V = 653	0.7465
Go/No-Go total errors	0.102 (− 1.64 to 2.36 ± 0.123)	t(51) = 0.82829	0.4114
Continuous Performance reaction time	− 1.013 (-4.19 to 1.60, ± 0.161)	t(52) = − 6.2899	<0.0001*
Continuous Performance false alarm errors	− 0.118 (− 5.08 to 1.04, ± 0.18)	V = 739	0.8387
Continuous Performance false miss errors	− 0.380 (− 3.71 to 0.73, ± 0.171)	V = 495	0.05146
Switching of Attention completion time	− 0.151 (− 3.23 to 1.96, ± 0.134)	t (49) = − 1.1306	0.2637
Switching of Attention errors	0.093 (− 2.57 to 0.94, ± 0.124)	V = 798	0.1225
Choice reaction time	0.22 (− 3.97 to 2.02, ± 0.14)	V = 865	**0.0113**
Memory Recognition immediate recall	0.493 (− 3.37 to 1.62, ± 0.111)	V = 1236	< 0.0001*
Memory Recognition delayed recall	0.133 (− 8.75 to 0.93, ± 0.188)	V = 1078	0.0004*
Digit Span	− 0.15 (− 4.90 to 2.20, ± 0.147)	V = 653	0.7465
Maze completion time	− 0.159 (− 3.05 to 1.93, ± 0.149)	t (49) = − 1.0683	0.2906
Maze total errors	− 0.031 (− 3.45 to 3.30, ± 0.171)	t (49) = − 0.18273	0.8558
Verbal Interference color word reading errors	0.076 (− 2.26 to 0.41, ± 0.073)	V = 1176	< 0.0001*
Verbal Interference color word reading reaction time	0.043 (− 1.74 to 1.65, ± 0.119)	t (51) = 0.36293	0.7182
Verbal Interference ink color naming errors	− 0.027 (− 3.06 to 1.13, ± 0.12)	V = 755	0.3911
Verbal Interference ink color naming reaction time	0.287 (− 2.45 to 2.02, ± 0.135)	t (49) = 2.1267	**0.0385**
Stroop interference by errors (ink color naming – color word reading errors)	− 0.096 (− 3.30 to 2.70, ± 0.132)	t (50) = − 0.72914	0.4693
Stroop interference by reaction time (ink color naming – color word reading reaction times)	0.236 (− 1.53 to 1.72, ± 0.102)	t (48) = 2.318	**0.0248**

^a^*p* values < .05 are indicated in bold text.

*Meets Bonferroni-corrected alpha threshold for statistical significance of *p* < 0.003.

### Relation of clutter underreporting to neurocognitive performance

We next explored neurocognitive performance measures as independent predictors of CIR-error using univariate linear regression (Table 4). Total errors on the Go/No-Go test (*β* =  − 0.12 (95% CI − 0.18 to − 0.05), *R*^*2*^ = 0.18, *p* = 0.002) predicted CIR-error. Differential reaction time for ink color naming vs color word reading on the Verbal Interference test (*β* = 0.13 (95% CI 0.04–0.22), *R*^2^ = 0.13, *p* = 0.010) also predicted CIR-error, however this relationship did not survive Bonferroni correction for multiple comparisons. Given the strong relationship between CIR-error and objective clutter, we additionally assessed neurocognitive variables when controlling for IE-CIR in a multivariate linear regression. This additionally suggested completion time for the Switching of Attention test (*β* =  − 0.06 (95% CI − 0.12 to − 0.01) *p* = 0.032) as a predictor of CIR-error at a trend level.

**Table 4:** Neurocognitive variables as predictors of clutter underreporting (CIR-error).

Neurocognitive Variables (N = 53)	As predictor of CIR-error	
	*β*	*p*^a^
Finger Tapping speed	0.008	0.759
Go/No-Go reaction time	− 0.032	0.627
Go/No-Go false alarm (no-go) errors	− 0.071	0.051
Go/No-Go false miss (go) errors	− 0.049	0.088
Go/No-Go total errors	− 0.115	0.002*
Continuous Performance reaction time	− 0.016	0.575
Continuous Performance false alarm errors	− 0.007	0.782
Continuous Performance false miss errors	− 0.046	0.089
Switching of Attention completion time	− 0.063	0.076
Switching of Attention errors	− 0.046	0.232
Choice reaction time	− 0.017	0.657
Memory Recognition immediate recall	− 0.019	0.654
Memory Recognition delayed recall	0.020	0.440
Digit Span	− 0.013	0.684
Maze completion time	− 0.015	0.658
Maze total errors	− 0.006	0.833
Verbal Interference color word reading errors	− 0.028	0.664
Verbal Interference color word reading reaction time	− 0.067	0.092
Verbal Interference ink color naming errors	0.041	0.319
Verbal Interference ink color naming reaction time	0.004	0.907
Stroop interference by errors (ink color naming – color word reading errors)	0.043	0.250
Stroop interference by reaction time (ink color naming – color word reading reaction times)	0.128	**0.010**

^a^*p* values < .0.05 are indicated in bold text.

*Meets Bonferroni-corrected alpha threshold of *p* < 0.003.

### Multivariate model

Considering all objective measures suggested as predictive of CIR-error, a multivariate linear model was tested incorporating objective clutter assessment (IE-CIR), Go/No-Go errors, Stroop interference by reaction time, and Switching of Attention completion time. This model revealed IE-CIR (*β* = 0.09 (95% CI 0.05–0.14), *p* < 0.001), Go/No-Go errors (*β* =  − 0.07 (95% CI − 0.13 to − 0.01), *p* = 0.03), and Stroop interference (*β* = 0.08 (95% CI 0.01–0.16), *p* = 0.03) to be significant independent predictors of CIR-error, and explained 50% of the variance of CIR-error measurements. IE-CIR (*β* = 0.10 (95% CI: 0.04–0.15), *p* = 0.002), Go/No-Go errors (*β* =  − 0.09 (95% CI − 0.15 to − 0.02), *p* = 0.014), and Stroop interference (*β* = 0.10 (95% CI 0.01–0.19), *p* = 0.043) remained significant independent predictors when included in a multivariate model controlling for age, gender, handedness, estimated IQ, depression, and use of psychotropic medication.

## Discussion

To our knowledge, our study is the first to assess anosognosia in HD in a manner that does not rely on direct subjective impressions of family or clinician raters and the first study to explore neurocognitive correlates of insight in HD. We found that most participants in our study underrated their clutter, that the degree of underrating was correlated with severity of clutter, and that underrating was correlated with behavioral performance on specific tests of neurocognitive function.

### Clutter rating discrepancies

Whether HD patients tend to underreport or overreport symptoms has been explored in prior studies^48,64^. Variable results when comparing self-ratings with other-ratings on diverse measures have suggested that such tendencies may be influenced by motivation or context (e.g., that participants seeking to enroll in studies might overestimate the severity of their disorder)^64^. In our study, however, the underreporting of clutter per self-CIR, performed in the context of screening for study participation, is inconsistent with such motivation. The correlations of this underreporting with objective clutter and with neurocognitive behavioral performance further argue against a purely social or contextual explanation for the discrepancies we observe.

Our study relies on discrepancies in CIR rating when the CIR is used as a self-report measure vs a clinician-rated measure. The close correlation of simultaneous self and clinician ratings reported in the literature suggests against gross differences in perception in individuals with HD. The time elapsed between asynchronous self- and IE-ratings in our study may suggest the possibility of interval change in clutter; yet discrepancies we observed were not correlated with inter-assessment interval. Deficits in memory or updating may explain discrepancies when HD participants perform self-rating outside of their homes, in line with observations that insight deficits in OCD are negatively correlated with verbal memory performance^65^ (though not consistently)^66^ or visual memory performance^66^. However, in our study, discrepancies in CIR rating were not predicted by behavioral measures of learning or memory.

The lower correlations of asynchronous (relative to synchronous) self and clinician clutter ratings observed in prior studies^48,62^ suggests that accuracy of clutter self-assessment, and indeed awareness itself, may be influenced by the presence of a third-party observer. This accords with what has been described in clinical literature, whereby individuals with HD may experience sudden increases in both awareness and distress when others enter their home^26^. Such fluctuating awareness in relation to third-party perspective may accord with findings from anosognosia in hemiplegia, schizophrenia, and dementia, in which unaware patients may be able to acknowledge deficits when exposed to evidence from a third-person perspective. This phenomenon has been modeled conceptually in dementia by invoking activation of distinct systems for autobiographical vs generic memory^67^.

### Anosognosia as characteristic of hoarding disorder

In our study, participants’ underreporting of clutter increased with objective clutter severity, the cardinal symptom of HD, suggesting that anosognosia may reflect core pathophysiological processes of HD. The relation between underreporting of clutter (insight impairment) and objective clutter severity (behavioral outcome) may find analogy in studies of insight in cocaine use disorder, where underreporting of desire to view cocaine-related images (relative to actual choice of such images in a laboratory context) predicted past-30-day cocaine use^68^. Importantly, clutter underreporting had no relation to self-reported clutter severity and was not correlated with self-report scales of HD severity, with the exception of a trend-level negative correlation with the Difficulty Discarding subscale of the SI-R. Absent or anti-correlations with self-report HD severity measures might be expected if underreporting is a true proxy of anosognosia. If anosognosia is common or characteristic of HD, however, this raises nosological questions about how the disorder might best be defined or assessed, i.e., whether to emphasize behavior and behavioral impacts (e.g., acquiring, saving, or clutter) or subjective distress associated with hoarding behaviors. The absence of a correlation between clutter underreporting and subscales of the SCI—which scores strength of agreement with various beliefs related to HD—is also notable, in as much as the best-validated instrument for assessing insight in other OCRDs, the BABS, emphasizes strength of disorder-related beliefs as fundamental to ratings of insight. If, as per prior literature, insight impairment in HD is a multi-dimensional construct with both ‘unawareness’ (anosognosia) and ‘delusionality’ as component axes^26,27^, our proxy measure appears to capture anosognosia more so than overvalued ideation or delusionality.

### Anosognosia and cognitive control

Errors on a Go/No-Go test of response inhibition most strongly predicted clutter underreporting in our sample. We are the first study to our knowledge to report Go/No-Go performance as a correlate of clinical insight, although in a neuroimaging study, decreased Go/No-Go task-based activations of cingulate and prefrontal cortex have been associated with unawareness of deficits in Alzheimer’s Disease^69^. As a measure of response inhibition, however, our finding is strongly consistent with replicated findings from research in psychotic disorders correlating deficits in clinical insight with deficits in Wisconsin Card Sort Test performance, and with the suggestion, based on this work, that insight impairment might reflect deficits in cognitive control and a tendency to perseverative error^17^. We additionally identified differential response time on a Stroop color/word task as a predictor of clutter underreporting. Correlations between Stroop performance and clinical insight impairment have been reported for OCD^65^ and for bipolar disorder^70^, on which basis insight impairment has similarly been suggested to reflect deficits in response inhibition and the processing of conflictual information. For our participants, we observed that increased clutter underreporting predicted better normalized response time performance on the ink color naming task relative to the word reading task. One speculative consideration is that insight deficits in OCRDs may correspond to deficits in the fast, ventral-stream visual pathway required for word recognition and logographic processing. Deficits in such ventral-stream processing for complex figures have been observed to correlate with insight impairment in body dysmorphic disorder^71,72^. Such deficits, if present in our HD participants, might be expected to slow response in the non-interference phase of our Stroop paradigm—which relies on fast reading of written color names—and yet speed performance in the interference phase of our Stroop paradigm, in as much as ‘overlearned’ word recognition might less potently interfere with ink color naming. Lastly, while below threshold for significance when included in a model with other clinical and neurocognitive predictors, we observed a trend toward impaired performance on the Switching of Attention test as a correlate of clutter underreporting, particularly when controlling for level of clutter. This is consistent with findings from OCD^66,73^, anorexia nervosa^74^, and bipolar disorder^70,75^, in which relative deficits in the Trail Making Test Part B have been found to correlate with impaired clinical insight. The observation that abnormalities of response inhibition, interference processing, and attention switching might correlate with insight impairment across diagnoses supports a general model in which awareness is intrinsically related to cognitive control. Conceptually, a theory of conscious awareness suggests that subjective awareness is a schematic model of attention itself, evolved to facilitate top-down control of attention^76^. Similarly, the CAM model for metacognitive awareness in dementia and other neurologic conditions suggests that deficits in executive error processing—required for the updating of self-concept based on sensory input—may present one pathway for the development of anosognosia^23^. Our data suggesting that pathological unawareness in HD correlates with deficits in cognitive control might provide a novel line of evidence in support of these general models.

### Limitations and future directions

Limitations of the current study include its exploratory nature and the possibility of Type I error. Additionally, our help-seeking sample may not be representative of the broader population of individuals with HD in terms of clinical insight: our participants lived in homes that were deemed non-squalorous and safely accessible for research staff, they were predominantly older women, and half were using psychotropic medication. While we suggest CIR-error as a face-valid proxy for anosognosia in HD, as a measure of clinical insight impairment more broadly it may not be fully content-valid, in that it may not capture clinically important dimensions of insight impairment in HD, such as the tendency to misjudge the value of objects or risks of clutter, or propensity to interpersonal distortions, by which individuals offering help may be perceived as threatening^26,27^. We additionally do not assess test–retest reliability of our measure. Nonetheless, the fact that CIR-error correlates with a measure of behavioral severity (objective clutter) and with measures of neurocognitive function identified as correlates of insight impairment in other disorders supports the construct validity of CIR-error as a proxy for anosognosia as a component of insight impairment.

Future work could assess whether clutter underreporting correlates with neurophysiologic or neuroimaging measures of error signaling known to be aberrant in OCD^77,78^ and HD^46,47^. In particular, in as much as HD—in contrast to OCD—may be associated with diminished, rather than exaggerated error signaling^46,47^, it would be of interest to test whether clutter underreporting correlates negatively with error signaling. This might replicate associations between insight impairment and hypoactive error signaling seen in cocaine use disorder^19^ or proposed in anosognosia for hemiplegia^18^. Given the prevalence, morbidity, and adverse prognostic significance of insight impairment across the spectrum of neuropsychiatric illness, and given the paucity of treatment interventions specifically targeting this dimension of illness, identifying the neural basis for insight in HD and other disorders will be critical to developing more effective future treatments for mental health conditions.

## Data Availability

The data that support the findings of this study are available on request from the corresponding author. The data are not publicly available, as they contain information that could compromise the privacy of research participants.

## Abbreviations

BABS: Brown Assessment of Beliefs Scale
CI: Confidence interval
CIR: Clutter Image Rating
DSM-5: Diagnostic and Statistical Manual of Mental Disorders, Fifth Edition
HD: Hoarding disorder
HDRS: Hamilton Depression Rating Scale
IE: Independent evaluator
OCD: Obsessive–compulsive disorder
OCRD: Obsessive–compulsive and related disorder
SCI: Saving Cognitions Inventory
SCID: Structured Clinical Interview for DSM-5
SD: Standard deviation
SI-R: Saving Inventory-Revised
SI-R-DD: Saving Inventory Revised, Difficulty Discarding subscale
Y-BOCS: Yale-Brown Obsessive Compulsive Scale
